# Drug–disease association prediction with literature based multi-feature fusion

**DOI:** 10.3389/fphar.2023.1205144

**Published:** 2023-05-22

**Authors:** Hongyu Kang, Li Hou, Yaowen Gu, Xiao Lu, Jiao Li, Qin Li

**Affiliations:** ^1^ Department of Biomedical Engineering, School of Life Science, Beijing Institute of Technology, Beijing, China; ^2^ Institute of Medical Information, Chinese Academy of Medical Sciences and Peking Union Medical College, Beijing, China

**Keywords:** drug repositioning, drug, disease, association prediction, literature, multi-feature fusion

## Abstract

**Introduction:** Exploring the potential efficacy of a drug is a valid approach for drug development with shorter development times and lower costs. Recently, several computational drug repositioning methods have been introduced to learn multi-features for potential association prediction. However, fully leveraging the vast amount of information in the scientific literature to enhance drug-disease association prediction is a great challenge.

**Methods:** We constructed a drug-disease association prediction method called Literature Based Multi-Feature Fusion (LBMFF), which effectively integrated known drugs, diseases, side effects and target associations from public databases as well as literature semantic features. Specifically, a pre-training and fine-tuning BERT model was introduced to extract literature semantic information for similarity assessment. Then, we revealed drug and disease embeddings from the constructed fusion similarity matrix by a graph convolutional network with an attention mechanism.

**Results:** LBMFF achieved superior performance in drug-disease association prediction with an AUC value of 0.8818 and an AUPR value of 0.5916.

**Discussion:** LBMFF achieved relative improvements of 31.67% and 16.09%, respectively, over the second-best results, compared to single feature methods and seven existing state-of-the-art prediction methods on the same test datasets. Meanwhile, case studies have verified that LBMFF can discover new associations to accelerate drug development. The proposed benchmark dataset and source code are available at: https://github.com/kang-hongyu/LBMFF.

## 1 Introduction

In recent decades, drug discovery techniques and biological systems have been intensively studied by multidisciplinary researchers. However, drug development remains a time-consuming, costly and labor-intensive process. It normally requires more than one to two billion dollars and an average of about 10–15 years to discover a new drug (Berdigaliyev and Aljofan, 2020). Approximately 90% of experimental candidates fail to pass the clinical trials (Mullard, 2022; Sun et al., 2022), owing to the unpredictable adverse reactions from new molecular structures. Drug repositioning (commonly known as “reuse of old drugs”) is a strategy for identifying new uses for approved or investigational drugs that are outside the scope of the original medical indications (Pushpakom et al., 2019; Schcolnik-Cabrera and Juárez-López, 2021). It can facilitate the drug development process, shorten the required time to 6.5 years and reduce the cost to 300 million dollars (Nosengo, 2016; Breckenridge and Jacob, 2019). From a drug safety perspective, repositioning drug candidates that have already passed early-stage clinical trials can sufficiently reduce the risk of failure.

In recent years, computational drug repositioning methods (Wu et al., 2013; Chan et al., 2019; Deng et al., 2022) have attracted continuous attention with explosive growth of large-scale genomic and phenotypic data. A variety of studies have confirmed the availability and desirable performances of computational drug repositioning (Giuliani et al., 2018; Piplani et al., 2021; Firoozbakht et al., 2022; Huang et al., 2022). Previous typical computational approaches include, but are not limited to, the following three: complex network methods, machine learning methods, and deep learning methods. In addition, knowledge organization methods have recently been gradually applied to the study of drug-disease relationship prediction.

Complex network methods refer to linking drugs to diseases through heterogeneous networks construction (Holzinger and Ritchie, 2012) with high-throughput omics data calculation (e.g., similarity calculation (Meng et al., 2021)). Network-based algorithms (e.g., random walk) have been demonstrated effective in drug-disease association prediction based on the topological characteristics in these heterogeneous networks. Wu et al. (2013) considered not only gene features, but also pathway, phenotype, biological process and other features in KEGG (2023) database to build a weighted disease-drug heterogeneous network, and predicted all possible drug-disease pairs through a clustering algorithm; Cami et al. (2013) constructed Predictive Pharmacointeraction Networks (PPINs) together with intrinsic and taxonomic properties of drugs and adverse events for drug-disease association prediction. Not limited to binary networks, Wang et al. (2014) calculated similarities through an iterative algorithm based on a three-layer heterogeneous graph of drugs, diseases and targets called TL-HGBI. Luo et al. (2016) first integrated comprehensive similarities of drugs and diseases and then identified potential indications of drugs with a double random walk method (MBIRW). In the follow-up study (Luo et al., 2019), they added phenotypes and genes into an upgraded drug repositioning recommendation system (DRRS) to predict novel drug indications with improved accuracy.

Machine learning methods have been established techniques in drug repositioning in recent years, which can be divided into two steps: first extracting biological features of drugs and diseases and then predicting novel drug-disease associations. Gottlieb et al. (2011) integrated multiple drug and disease similarity measurements and sorted predicted drug-disease pairs by logistic regression algorithm, which can be applied to large-scale data. Support vector machine (Wang et al., 2013) and random forest (Kim et al., 2019) are also considered brilliant methods for drug-disease association predictions and achieved good performance in early studies. Napolitano et al. (2013) reported a joint kernel based on drug-related data, such as gene expression, chemical structure and target information, in support vector machine classification to predict drug repositioning. Machine learning approaches are effective in integrating prior information. However, its biological interpretability is limited (Shah et al., 2021) and the performance is constrained by the sparsity of biological interactions. Also, due to the complexity of matrix operations, processing large-scale data is highly challenging.

The remarkable rise of deep learning has led to an overwhelming amount of new research. Long Short-Term Memory (Lyu et al., 2017), Bidirectional Encoder Representation from Transformers (Lee et al., 2020) and Graph Neural Network have provided significant improvements in biomedical information retrieval (Sun et al., 2021), question and answer systems (Wen et al., 2020) and image recognition (Vellal et al., 2021). In addition, several studies have described the use of these techniques for drug discovery. Zitnik et al. (2018) presented a graph convolution neural network to handle multimodal graphs with a large number of edge types including drug, protein, target and side effect. Fatehifar amd Karshenas (2012) proposed a BI-LSTM model and Pang et al. (2022) proposed a novel attention-mechanism-based multidimensional feature encoder to extract the drug-drug interaction, which performed better than some state-of-the-art methods. Li et al. (2020) acquired potential feature representations from miRNA and disease similarity network with graph convolutional network and developed a Neural Inductive Matrix Completion method for miRNA-disease association prediction. Graph Neural Network (GNN) (2023) performs particularly well in handling comprehensive information and heterogeneous semantically-rich graphs. The existing GNN processing methods (Wu et al., 2021) contain Graph Convolutional Network, Graph Sample and Aggregate, Graph Attention Network, *etc.* With the rapid accumulation of biological network data, GNN has become an effective tool in bioinformatics tasks (Zhang et al., 2021). Taking drug development as an example, it has been proven a practical way of achieving greater efficiency in drug attribute prediction (Gu et al., 2021), drug side effect prediction (Zitnik et al., 2018), relationship extraction (Al-Sabri et al., 2022), *etc.*

Ontology (Bandrowski et al., 2016) and knowledge graph (Nicholson and Greene, 2020) can provide structured, computable organization and management of large amounts of data. Several biomedical ontologies have been proven useful in biomedical text mining studies (Shen and Lee, 2016; Kafkas and Hoehndorf, 2019), including Disease Ontology (2023), Human Phenotype Ontology (HPO) (2023), UMLS, *etc.* Different ontologies can also be constructed based on their research objectives. Brown and Patel (2017) mined drug-drug links by mapping drug terminology to standardized terms from MeSH. Karim et al. (2019) extracted attribute and relationship embedding from a drug-adverse reaction knowledge graph they developed to infer drug-drug interactions according to biomedical databases and literature. Moon et al. (2021) constructed a knowledge graph to learn drug-disease-target embedding to inform drug repurposing hypotheses.

In addition to the drug-disease associations proved by clinical practice, physicians and researchers have conducted further studies and explorations into new drug combinations and drug indications. They detailed the entire process and reported it in a timely manner in the form of scientific literature. Compared with public databases such as Drugbank (2023), PharmGKB (2023), and MeSH (2023), *etc.*, biomedical literature contains not only a massive number of biomedical entities (Chen et al., 2020), such as drugs, indications, side effects, and targets, but also associations have been discovered recently. Considering the vast amount of semantic information contained in scientific literature, current approaches need to improve the integration ability of validated relational features in public databases presented as structured data along with the newly discovered relational features and semantic features s in biomedical scientific literature.

To overcome the mentioned limitation, we proposed a novel drug-disease association prediction method called Literature Based Multi-Feature Fusion (LBMFF). LBMFF not only integrated multiple heterogeneous biological interactions (drug, disease, side effect and target), but also extracted semantic embeddings and contextual information from large-scale of scientific literature. Specifically, we constructed drug-drug similarities and disease-disease similarities based on multi-feature and associations from public databases and PubMed literature. Then, a GCN with an attention mechanism was employed to capture structural information from a comprehensive similarity matrix and known drug-disease associations. LBMFF achieved optimal results compared to single-feature methods, which demonstrated the significance of literature information and feature fusion. It also showed superior performance in drug-disease association prediction compared to 7 state-of-the-art methods.

## 2 Materials and methods

### 2.1 Dataset

In our study, the benchmark dataset downloaded from Zhang et al. (2018), contains 269 drugs, 598 diseases and 18,416 drug-disease associations originated from Comparative Toxicology Database (CTD). What’s more, we extracted drug chemical structures (represented by SMILES) and drug-target associations from Drugbank, drug-side effect associations from SIDER (2023) and diseases tree numbers from MeSH as multi-features for drug-drug similarities and disease-disease similarities calculation. Overall, in addition to the raw data from CTD, we extended the benchmark dataset to 269 drug SMILES sequences, 3,797 side effects and 43,508 drug-side effect associations, 266 targets and 722 drug-target associations.

More importantly, we searched and selected 673,665 full-text scientific literature, which titles or abstracts contained the drugs or diseases from the benchmark dataset. This vast literature serves as a corpus for the semantic similarity computation section based on a pre-training and fine-tuning BERT model.

Furthermore, we introduced a dataset from Wang et al. (2014), named TL-HGBI, for method portability validation. It contains 963 drugs, 1,263 diseases and 54,921 drug-disease associations originating from CTD. Similarly, we also collected drug SMILES sequences, disease MeSH tree numbers, drug-side effect associations, drug-target associations and scientific literature.

### 2.2 Architecture of LBMFF

**FIGURE 1:**
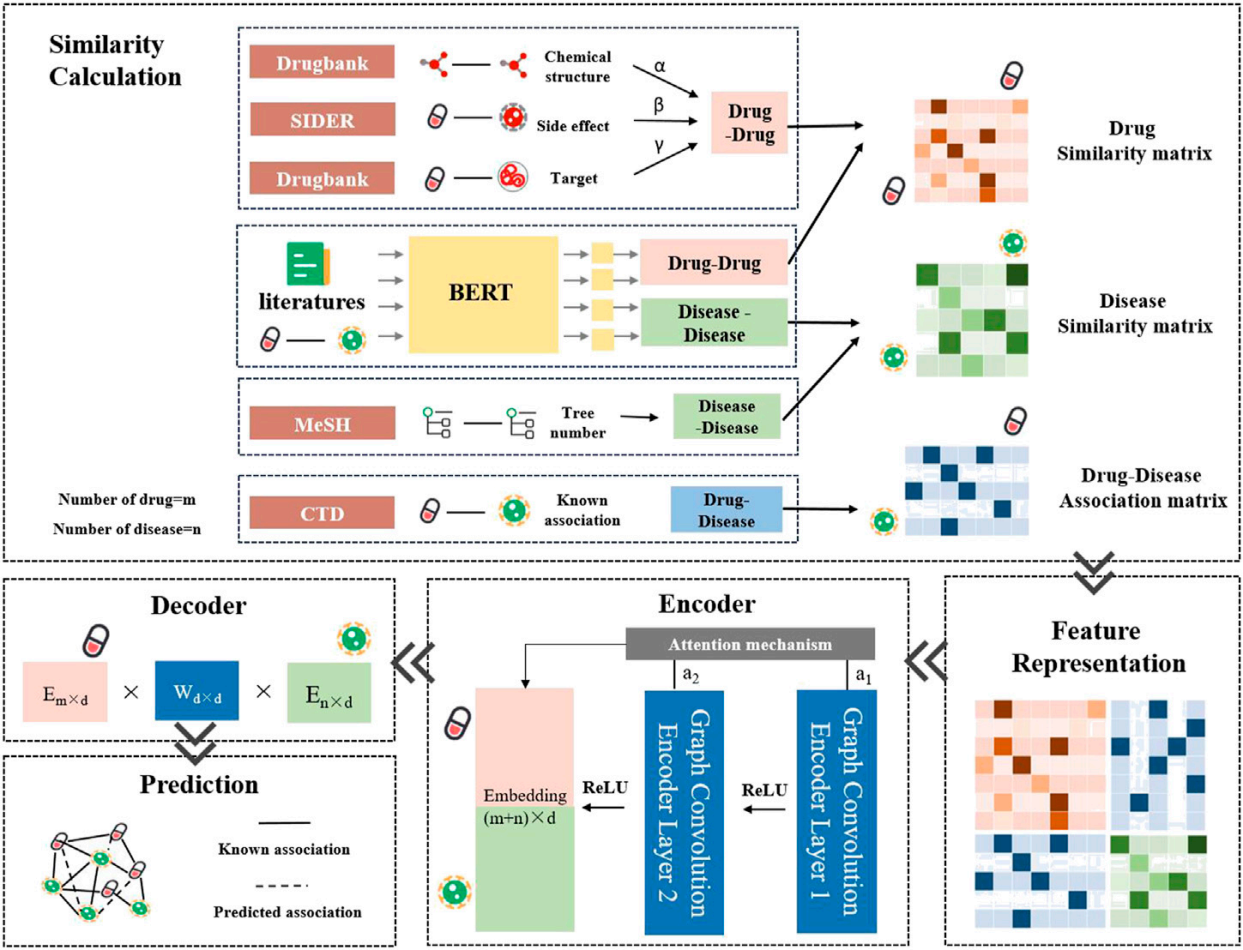
The workflow of LBMFF.

The LBMFF combined embeddings in drug-disease-target-side effect networks from public databases including CTD, Drugbank, SIDER and MeSH. What’s more, semantic features from a vast amount of scientific literature were added to LBMFF as an improved approach. The workflow of LBMFF was briefly shown in Figure 1.

• Association and Semantic Feature Extraction. We integrated several measurements for drug and disease similarities computing, including drug SMILES sequences, disease MeSH tree numbers, drug-side effect associations, drug-target associations, and literature semantic information. A Pre-training and fine-tuning BERT model was introduced for semantic information recognition and understanding.
• Similarity Calculation and Feature Representation. For feature fusion and similarity computing, an adjusted weight for each measurement was applied to achieve optimal performance by a step of 0.01. We then constructed a feature matrix based on the drug fusion similarity, disease fusion similarity and known drug-disease associations.
• Association Prediction. We applied two GCN layers to learn the embeddings of drugs and diseases with an attention mechanism. An inner product decoder was used to discover unknown drug-disease associations.

### 2.3 Feature extraction

#### 2.3.1 Drug chemical structure

Drugs can usually be characterized by biological or chemical descriptors, that is, molecular fingerprints. Molecular fingerprints are ways of encoding the structure of a molecule. The most common type of fingerprint is a series of binary digits (bits) that represent the presence or absence of particular substructures in the molecule (Cao et al., 2012). In this study, we adopt the drug SMILES sequences and generated their Morgan fingerprint to capture the molecular substructure and to calculate the chemical structure drug-drug similarities.

Based on this principle, we convert a drug into an n-dimensional fingerprint vector $X=\left[x_{1},x_{2},\ldots \ldots ,x_{n}\right]$ , where *n* is the number of all substructures. If there is a substructure in the drug, $x_{n}$ will be 1, otherwise, it will be 0. In this section, we adopt the Jaccard index to calculate drug-drug similarity:

Sijc=Xi∩XjXi∪Xj (1)

where $S_{ij}^{c}$ is the similarity between drug $r_{i}$ and drug $r_{j}$ , $X_{i}$ is an n-dimensional vector of drug $r_{i}$ , $X_{j}$ is an n-dimensional vector of drug $r_{j}$ .

#### 2.3.2 Drug-side effect interaction

From the view of “if a drug has the same side effects, it may have the same indication”, in 2008, Campillos et al. (2008) proposed a method to calculate drug similarity based on drug-side effect interaction, which has been widely used in subsequent studies. A k-dimensional drug vector $Y=\left[y_{1},y_{2},\ldots \ldots ,y_{k}\right]$ can be generated based on the known drug-side effect interaction from SIDER, where k is the number of related side effects. If there is an interaction between the drug and side effects, $y_{k}$ will be 1, otherwise, it will be 0. We also adopt the Jaccard index to calculate drug-drug similarity $S_{ij}^{s}$ on this dimension.

#### 2.3.3 Drug-target interaction

Similarly, drug-target interactions are also a valid approach for drug similarity calculations. We extract this information from Drugbank and adopt the Jaccard index to measure drug-drug similarity $S_{ij}^{t}$ .

#### 2.3.4 MeSH semantic attribute for disease

The Medical Subject Headings (MeSH) thesaurus is a controlled and hierarchically-organized vocabulary produced by the National Library of Medicine. Wang et al. (2010) proposed a disease semantic similarity method by using MeSH hierarchically organized information, which was regarded as a directed acyclic graph (DAG). For the disease *d*, we denote its DAG as $DAG\left(d\right)=\left(N\left(d\right),\varepsilon \left(d\right)\right)$ , where $N\left(d\right)$ is a node set including disease *d* and its ancestor nodes, $\varepsilon \left(d\right)$ is the set of direct links from parent nodes to child nodes in $N\left(d\right)$ . We define the semantic value of disease *d* as $DV\left(d\right)=\sum_{n\in N\left(d\right)}C_{d}\left(n\right)$ , where semantic contribution decay factor $C_{d}\left(n\right)$ can be formulated as:

$$C_{d}\left(n\right)=\left\{\begin{array}{c} 1\;if\;n=d\\ \max\left\{0.5*C_{d}\left(n^{\prime }\right)|n^{\prime }\in children\;of\;n\right\}\;if\;n\neq d \end{array}\right.$$ (2)

Based on this definition, disease semantic similarity can be presented as follow:

$$S_{ij}^{d}=\frac{\sum_{n\in N\left(d_{i}\right)\cap N\left(d_{j}\right)}\left(c_{d_{i}}\left(n\right)+c_{d_{j}}\left(n\right)\right)}{DV\left(d_{i}\right)+DV\left(d_{j}\right)}$$ (3)

where $S_{ij}^{d}$ is the similarity between disease $d_{i}$ and disease $d_{j}$ . Intuitively, two diseases with more ancestor nodes tend to have a higher semantic similarity.

#### 2.3.5 Literature semantic similarity based on BERT

BERT (2023), which represents Bidirectional Encoder Representations from Transformers, is based on a multi-layer bidirectional Transformer model in which every output element is connected to every input element, and the weightings between them are dynamically calculated based upon their connection. The transformer mechanism gives BERT its increased capacity to understand context and ambiguity in language.

**FIGURE 2:**
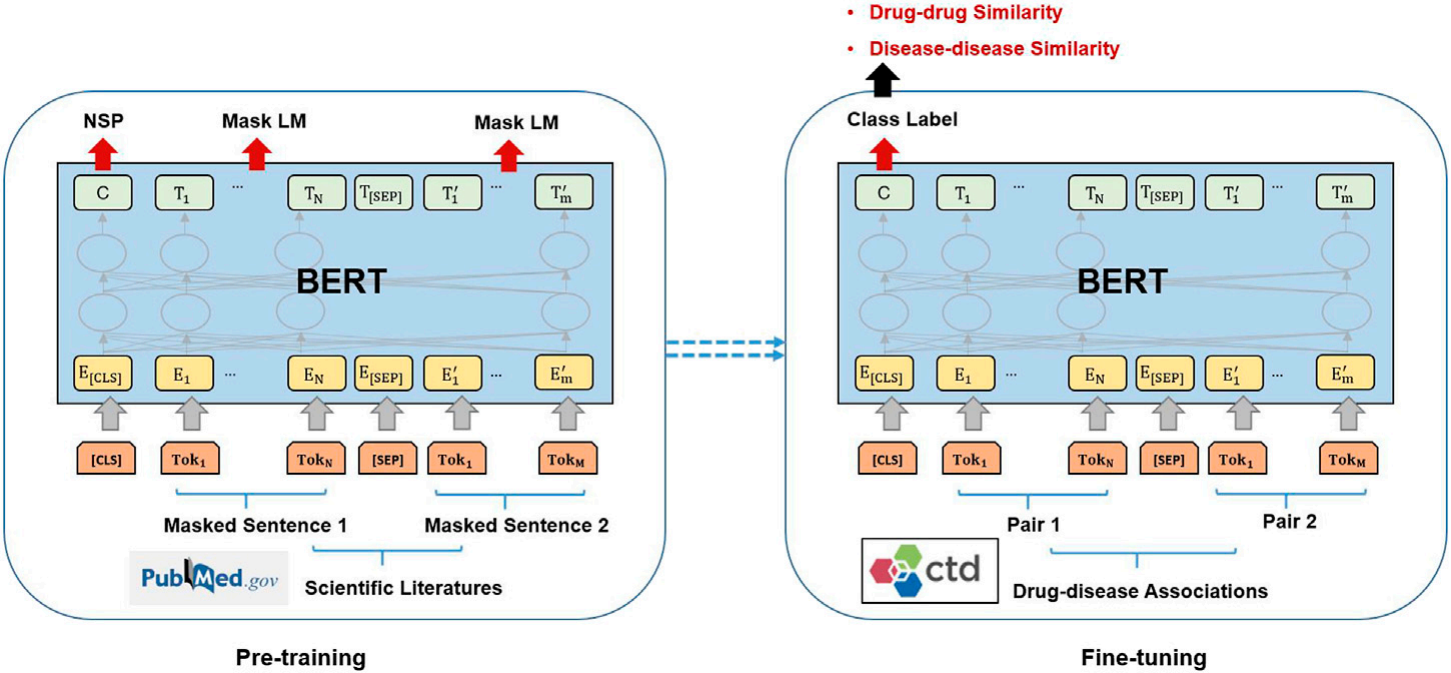
Pre-training and fine-tuning BERT.

The model architecture was shown in Figure 2.

The BERT model is firstly pre-trained on the dataset containing 673,665 full-text scientific literature downloaded from PubMed, which includes drugs and diseases mentioned in the benchmark dataset. We implement parameters during the pre-training step, with training epochs of 10,000, vector dimension of 128, learning rate of 0.01and dropout of 0.1.

After pre-training the BERT model, we used fine-tuning to train a binary classification model via five-fold cross validation. Fine-tuning is a method of making small adjustments to a pre-trained model for a specific task. In the binary classification task, our objective was to minimize the cross-entropy loss function, which can be represented as following:

$$Loss=-y\log\left(p\right)-\left(1-y\right)\log\left(1-p\right)$$ (4)

where y is the label (in our case, either 0 or 1), and p is the predicted probability of the label being 1. The cross-entropy loss function is used to measure the difference between the predicted probability distribution and the true label, allowing us to optimize the model’s parameters to minimize error. During the fine-tuning process, we used the training dataset to adjust the BERT model’s parameters, improving the model’s classification accuracy. Using this approach, we were able to apply the BERT model to predict associations between drugs and diseases and calculate similarities between drug-drug and disease-disease pairs. In this binary classification task, when a drug is associated with a disease, it is 1. Otherwise, it is 0.

The training procedure uses only drug-disease associations from the training set and no associations from the test set. Specifically, we use this fine-tuning process to improve the accuracy of semantic comprehension and ability of similarity calculation for drugs and diseases. When we feed drug-drug or disease-disease pairs into the BERT-model, it can compute the corresponding drug-drug similarities and disease-disease similarities (semantically similar to the binary classification task of associations) based on the previous mentioned fine-tuning BETT-model.

### 2.4 Similarity matrix fusion

Based on the above four characteristics (chemical structure, drug-side effect, drug-target, literature-based semantic representation) of drugs, drug similarity $S_{ij}^{r}$ between drug $r_{i}$ and drug $r_{j}$ based on multi-feature fusion can be expressed as:

$$S_{ij}^{r}=\alpha S_{ij}^{c}+\beta S_{ij}^{s}+\gamma S_{ij}^{t}+\delta_{1}S_{ij}^{l\left(r\right)}$$ (5)

where $\alpha +\beta +\gamma +\delta_{1}=1$ . The optimization step for the combination is 0.01.

Similarly, disease similarity $S_{ij}^{d}$ between disease $d_{i}$ and disease $d_{j}$ based on MeSH and literature semantic features can be expressed as:

$$S_{ij}^{d}=\theta S_{ij}^{d}+\delta_{2}S_{ij}^{l\left(d\right)}$$ (6)

where $\theta +\delta_{2}=1$ .

### 2.5 Feature representation

In this paper, we construct the association feature representation between drug and diseases based on drug comprehensive similarity matrix, disease comprehensive similarity matrix and known drug-disease associations. Binary matrix $A\epsilon {\left\{0,1\right\}}^{m*n}$ represents drug-disease associations, where *m* is the number of drugs and *n* is the number of diseases. When drug $r_{i}$ is associated with disease $d_{j}$ , $A_{ij}=1$ ; otherwise, $A_{ij}=0$ .

The adjacency matrix of drug-disease association features can be expressed as:

$$A_{H}=\left[\begin{array}{cc} ∼S^{r} & A\\ A^{T} & ∼S^{d} \end{array}\right]$$ (7)

where $∼S^{r}$ and $∼S^{d}$ are the normalization matrix of comprehensive similarity matrix $S^{r}$ and $S^{d}$ through Laplace transformation.

$$∼S^{r}=D_{r}^{-\frac{1}{2}}S^{r}D_{r}^{-\frac{1}{2}}$$ (8)

$$∼S^{d}=D_{d}^{-\frac{1}{2}}S^{d}D_{d}^{-\frac{1}{2}}$$ (9)

where $D=diag\left(\sum_{j}S_{ij}\right)$ is the degree matrix.

### 2.6 Associated prediction based on GCN

GCN is a multilayer connected neural network architecture used to learn low-dimensional representations of nodes from graph-structured data (Thomas and Kipf, 2017).

#### 2.6.1 Encoder

The adjacency matrix $A_{H}$ mentioned above is introduced into the GCN encoder to extract drug and disease embeddings respectively. We initialize the embeddings of drugs and diseases as $H^{\left(0\right)}=\left[\begin{array}{cc} 0 & A\\ A^{T} & 0 \end{array}\right]$ , and set the associations in the test dataset to 0. Subsequently, the GCN layer is denoted as:

$$H^{\left(l+1\right)}=f\left(H^{\left(l\right)},A\right)=ReLU\left({\tilde{D}}^{-\frac{1}{2}}A{\tilde{D}}^{-\frac{1}{2}}H^{\left(l\right)}W^{\left(l\right)}\right)$$ (10)

where $H^{\left(l+1\right)}$ is the embedding at the lth-layer, $\tilde{D}$ is the degree matrix of $A_{H}$ , $W^{\left(l\right)}$ is a trainable weight matrix of the lth-layer. At the same time, we use ReLU as an activation function.

#### 2.6.2 Decoder

Furthermore, we introduce a layer attention mechanism (Thomas and Kipf, 2017) in LBMFF to fully utilize the drug and disease embedding and adaptively adjust their importance weights dynamically of different GCN layers.

The final embedding of drugs and diseases is denoted as follows:

$$E=\left[\begin{array}{c} E_{r}\\ E_{d} \end{array}\right]=\sum_{l}a_{l}H^{\left(l\right)}$$ (11)

where $E_{r}\in R^{m*d}$ is the final embedding of drugs, $E_{d}\in R^{n*d}$ is the final embedding of drugs. $a_{l}$ is a weight initialized as 1l+1 and auto-learned by neural networks thereafter.

To reconstruct the adjacency matrix for drug-disease associations, we introduce sigmoid as the activation function into the GCN decoder, and the predicted association matrix can be expressed as:

$$A^{\prime }=sigmoid\left(E_{r}∙W∙E_{d}\right)$$ (12)

where $A_{ij}^{\prime }$ is the associated prediction score of drug $r_{i}$ and disease $d_{j}$ , $W\in R^{d*d}$ is the trainable parameter matrix.

Due to the fast training speed of the GCN model, it can be retrained when new drug/disease nodes were added.

## 3 Result and discussion

### 3.1 Experimental setup and performance evaluation

We constructed five-fold cross validation to evaluate the performance of LBMFF in our study. All known drug-disease associations were randomly divided into five mutually exclusive subsets of the same size, that is, four subsets were selected as the training set each time, while the remaining one was used as the test set. Each round of training started from the initial state and the association prediction was performed on the test set after training. At last, we adopted the average of the five training performances as the final results.

Area Under Curve (AUC) and Area Under Precision/Recall Curve (AUPR) were used as the primary metrics to evaluate the prediction performances. In addition, we also take several binary classification metrics into consideration, including accuracy (Acc), recall (Rec), specificity (Spe), precision (Pre) and F1-score (F1).

### 3.2 Performances of literature based multi-feature fusion (LBMFF)

In this study, we considered multi-features of drugs and diseases, which were chemical structure, drug-side effect association, drug-target association, disease similarity from MeSH, and especially semantic similarity supported by a large scale of literature. The weights of each feature were optimized at a step of 0.01 during the process of feature fusion. The optimal prediction results appeared with the fusion coefficients of $\alpha =0.08,\beta =0.16,\gamma =0,16,\delta_{1}=0.60;\theta =0.40,\delta_{2}=0.60$ . Our predictive model achieved AUC and AUPR of 0.8743 and 0.5694 in these cases.

### 3.3 Ablation study

To demonstrate the significance of the vast amount of literature texting mining for association prediction, we did ablation studies that compared LBMFF with single-feature methods and Multi-Feature Fusion (MFF) method in Table 1.

**TABLE 1:** Algorithm performance Comparison between multi-attribute fusion and single attribute.

Methods	Features					AUPR	AUC	F1	Acc	Rec	Spe	Pre
	Chemical structure	Target	Side effect	Literature Semantic								
1	√					0.5799 ± 0.001	0.8711 ± 0.003	0.5541 ± 0.017	0.8904 ± 0.000	0.5907 ± 0.002	**0.9294 ± 0.008**	0.5220 ± 0.000
2		√				0.5852 ± 0.000	0.8707 ± 0.001	0.5598 ± 0.002	**0.8910 ± 0.016**	0.6007 ± 0.000	0.9289 ± 0.036	**0.5244 ± 0.021**
3			√			0.5787 ± 0.001	0.8688 ± 0.005	0.5534 ± 0.003	0.8847 ± 0.002	0.6194 ± 0.000	0.9193 ± 0.020	0.5009 ± 0.002
4				√		0.5928 ± 0.002	0.8779 ± 0.000	0.5644 ± 0.021	0.8934 ± 0.003	0.5986 ± 0.013	0.9277 ± 0.001	0.5242 ± 0.056
MFF	√	√	√			0.5897 ± 0.002	0.8769 ± 0.002	0.5623 ± 0.030	0.8904 ± 0.000	0.6095 ± 0.001	0.9270 ± 0.005	0.5230 ± 0.0003
LBMFF	√	√	√	√		**0.5961 ± 0.001**	**0.8818 ± 0.003**	**0.5655 ± 0.001**	0.8885 ± 0.001	**0.6287 ± 0.005**	0.9224 ± 0.000	0.5154 ± 0.031

The best results are in bold faces and the second-best results are underlined.

Firstly, it should be noted the significance of literature-based semantic feature on the prediction of unknown relations compared to other single-feature methods, with the most relative improvements of 2.4% on AUPR. Secondly, the results also indicated that MFF outperforms the single-feature methods in terms of AUPR, AUC and Acc. Thirdly, LBMFF reached the best performance: AUC = 0.8818, which achieved relative improvements of 1.23%, 1.28%, 0.49%, 0.56% higher than single-feature methods and 0.56% higher than MFF; AUPR = 0.5961, achieving relative improvements of 2.79%, 1.87%, 3.02%, 0.44% higher than single-feature methods and 1.09% higher than MFF. We reached the best performance in both F1 and Rec.

According to the aforementioned performance metrics, MFF first achieved better performance than single-feature methods due to the adjusted weights for each measurement. It demonstrated that feature fusion played an essential role in drug-disease association prediction due to the integrated information from different dimensions. LBMFF further extracted semantic information from large-scale literature, which then led to improved performance of MFF-based model.

### 3.4 Comparison with state-of-the-art methods

In this section, we compared LBMFF with seven state-of-the-art association prediction methods by using the same dataset, Bdataset:

Specifically, we listed these methods as follows.

• BNNR (Yang et al., 2019) was a bounded nuclear norm regularization method carried out on an adjacency matrix of a heterogeneous drug-disease network.
• DRHGCN (Cai et al., 2021) and DRWBNCF (Meng et al., 2022) partly used GCN-based, deep-learning methodology and weighted bilinear neural collaborative filtering based on heterogeneous information fusion for the drug repositioning approach.
• LAGCN (Yu et al., 2022) predicted drug-disease associations through a layer attention graph convolutional network.
• NIMCGCN (Li et al., 2020) was a novel method of neural inductive matrix completion with GCN for miRNA-disease association prediction.
• DDA-SKF (Gao et al., 2021) constructed multiple similarity kernels for drugs and diseases, and the Laplacian regularized least squares algorithms were used to obtain the association matrix.
• REDDA (Gu et al., 2022) proposed a general heterogeneous GCN-based node embedding block, a topological subnet embedding block, a graph attention block, and a layer attention block.

**FIGURE 3:**
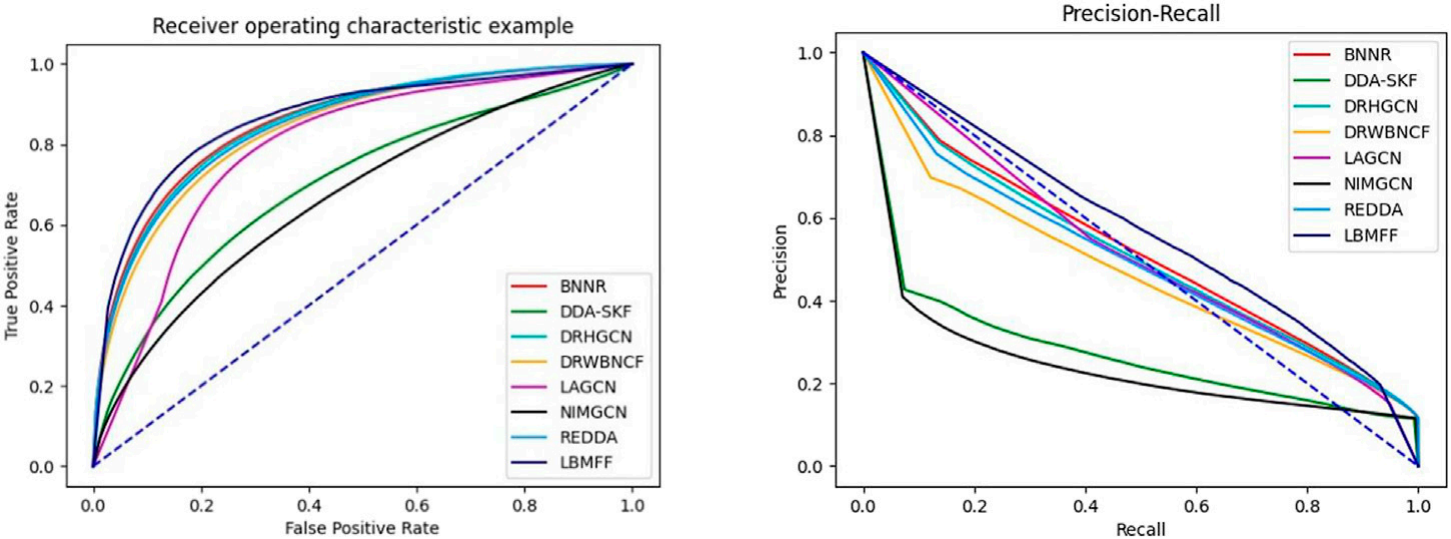
AUC and AUPR metrics of 8 methods.

According to Table 2; Figure 3, LBMFF achieved the best performance in terms of all the evaluation metrics. LBMFF achieved an AUC value of 0.8818, which was higher than the seven state-of-the-art methods with AUC values of 0.8561, 0.7006, 0.8529, 0.8045, 0.6684, 0.8375, and 0.8466. Meanwhile, our method significantly outperformed all baseline methods on AUPR. More specifically, LBMFF achieved an AUPR value of 0.5961 and achieved a relative improvement of 16.09% compared to the second-best result of 0.5135 from LAGCN. Focusing on the F1 and precision (Pre), our method had distinct advantages over all the baseline methods with relative improvements of 10.71% and 10.51% to the second-best results of BNNR. Even though our method achieved slightly better performance in terms of recall (Rec = 0.6287) and specificity (Spe = 0.9224) than the second-best results (Rec = 0.6005, Spe = 0.9166), these two evaluation metrics were significantly better than the other methods with the average relative improvements of 24.31% and 6.84%. We extracted multiple heterogeneous biological interactions and semantic embeddings to improve prediction accuracy. These results tended to indicate that LBMFF had a state-of-the-art performance against all baseline methods in novel drug-disease association prediction, owing to the superior integration ability of multi-feature from not only public databases and scientific literature.

**TABLE 2:** Performance compared with 7 baseline methods.

Methods	AURP	AUC	F1	Acc	Rec	Spe	Pre
BNNR	0.5166 ± 0.008	0.8561 ± 0.002	0.5108 ± 0.001	0.8761 ± 0.036	0.5649 ± 0.002	0.9164 ± 0.000	0.4664 ± 0.001
DDA-SKF	0.2521 ± 0.001	0.7006 ± 0.000	0.3281 ± 0.002	0.7900 ± 0.007	0.4478 ± 0.005	0.8342 ± 0.004	0.2591 ± 0.001
DRHGCN	0.5063 ± 0.002	0.8529 ± 0.004	0.5013 ± 0.000	0.8746 ± 0.001	0.5503 ± 0.022	0.9166 ± 0.006	0.4604 ± 0.002
LAGCN	0.5135 ± 0.000	0.8045 ± 0.002	0.4699 ± 0.005	0.7966 ± 0.000	0.6005 ± 0.008	0.8220 ± 0.052	0.4198 ± 0.002
NIMCGCN	0.2316 ± 0.004	0.6684 ± 0.000	0.2889 ± 0.007	0.7611 ± 0.026	0.4227 ± 0.001	0.8049 ± 0.003	0.2199 ± 0.006
DRWBNCF	0.4552 ± 0.035	0.8375 ± 0.020	0.4739 ± 0.001	0.8646 ± 0.001	0.5321 ± 0.000	0.9076 ± 0.002	0.4280 ± 0.000
REDDA	0.4903 ± 0.000	0.8466 ± 0.016	0.4936 ± 0.045	0.8693 ± 0.004	0.5562 ± 0.003	0.9098 ± 0.000	0.4440 ± 0.002
LBMFF	**0.5961 ± 0.001**	**0.8818 ± 0.003**	**0.5655 ± 0.001**	**0.8885 ± 0.001**	**0.6287 ± 0.005**	**0.9224 ± 0.000**	**0.5154 ± 0.031**

The best results are in bold faces and the second-best results are underlined.

**FIGURE 4:**
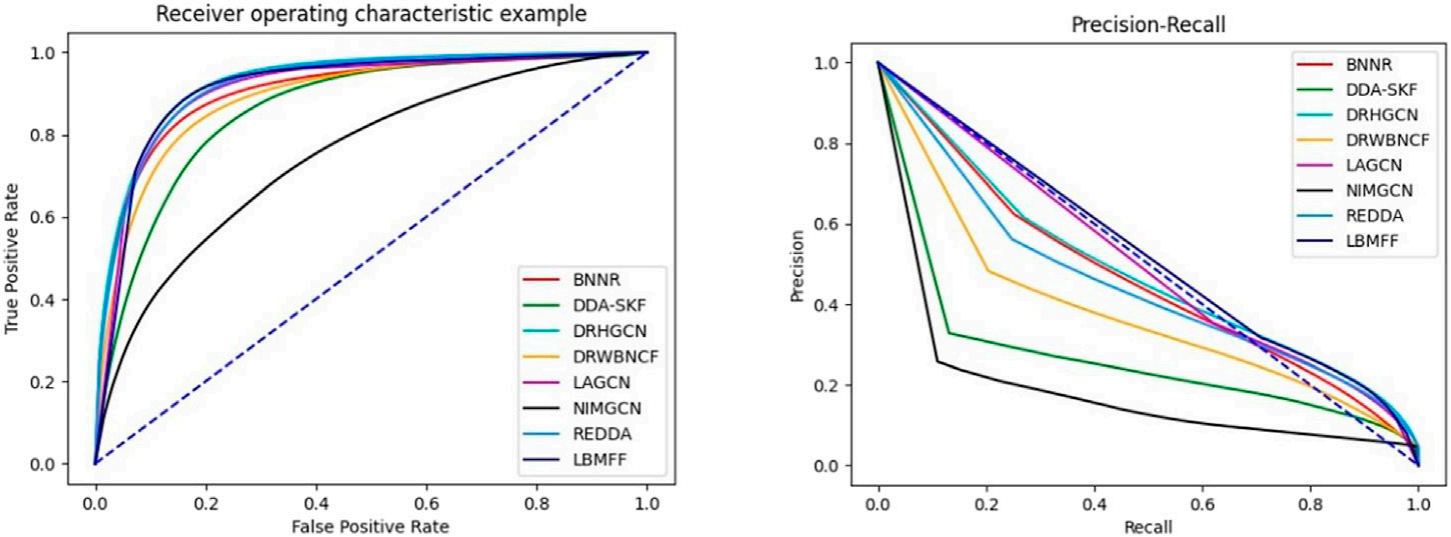
AUC and AUPR metrics of 8 methods on TL-HGBI dataset.

To further verify the effectiveness of LBMFF, we denoted another public benchmark, TL-HGBI with 963 drugs, 1,263 diseases and 54,921 drug-disease associations, into LBMFF and the seven baseline methods mentioned above. The results in Table 3; Figure 4 verified the superior predictive solidarity of our method. REDDA respectively achieved excellent performance measured by AURP, F1 and Rec. What’s more, AUPR was 5.86% higher than the second best method LAGCN.

**TABLE 3:** Performance compared with 7 baseline methods on TL-HGBI dataset.

Methods	AURP	AUC	F1	Acc	Rec	Spe	Pre
BNNR	0.4502 ± 0.002	0.9065 ± 0.001	0.4640 ± 0.003	**0.9462 ± 0.000**	0.5098 ± 0.020	**0.9671 ± 0.015**	**0.4261 ± 0.004**
DRHGCN	0.4824 ± 0.000	**0.9295 ± 0.032**	0.4723 ± 0.016	0.9442 ± 0.035	0.5459 ± 0.004	0.9633 ± 0.000	0.4161 ± 0.000
DRWBNCF	0.3432 ± 0.001	0.8927 ± 0.004	0.4013 ± 0.000	0.9306 ± 0.002	0.5090 ± 0.002	0.9508 ± 0.030	0.3314 ± 0.001
LAGCN	0.4970 ± 0.004	0.9155 ± 0.005	0.4586 ± 0.007	0.9413 ± 0.002	0.5440 ± 0.033	0.9603 ± 0.000	0.3968 ± 0.001
NIMCGCN	0.1532 ± 0.003	0.7490 ± 0.018	0.2317 ± 0.020	0.9012 ± 0.000	0.3265 ± 0.005	0.9287 ± 0.001	0.1802 ± 0.006
DDA-SKF	0.2266 ± 0.015	0.8608 ± 0.007	0.3136 ± 0.000	0.9071 ± 0.001	0.4646 ± 0.003	0.9283 ± 0.002	0.2368 ± 0.003
REDDA	0.4243 ± 0.000	0.9225 ± 0.001	0.4493 ± 0.013	0.9406 ± 0.001	0.5308 ± 0.002	0.9602 ± 0.025	0.3898 ± 0.010
LBMFF	**0.5261 ± 0.003**	0.9160 ± 0.000	**0.4821 ± 0.002**	0.9429 ± 0.003	**0.5898 ± 0.002**	0.9596 ± 0.005	0.4078 ± 0.007

The best results are in bold faces and the second-best results are underlined.

### 3.5 Case study

To demonstrate the capability of LBMFF to discover new indications and new therapies, all known drug-disease associations have been used to predict unknown drug-disease associations with this model. We conducted case studies with verification from clinical indications that were already in use, ClinicalTrials, CTD and public literature. ClinicalTrials is the largest clinical trials database run by the United States National Library of Medicine (NLM), holding registrations from over 329,000 trials from 209 countries. CTD is a publicly available research resource that curates scientific data describing relationships between chemicals, genes and human diseases by professional curators. In our study, we constructed three kinds of case studies to verify the predictive capability: 1) the top 10 drug-disease associations predicted by LBMFF in Table 4, 2 the top 10 associated diseases for given drugs predicted by LBMFF in Table 5, 3 the top 10 associated drugs for given diseases predicted by LBMFF in Table 6.

**TABLE 4:** Top 10 drug-disease associations.

No.	MESH ID	Drug Name	MESH ID	Disease Name	Evidence
1	C055162	Clopidogrel	D006973	Hypertension	ClinicalTrials/CTD
2	D002738	Chloroquine	D018771	Arthralgia	NA
3	D012293	Rifampin	D011014	Pneumonia	CTD/PMID: 28870736
4	D019808	Losartan	D001281	Atrial Fibrillation	ClinicalTrials/PMID: 25787020
5	D019821	Simvastatin	D010190	Pancreatic Neoplasms	ClinicalTrials/CTD/PMID: 32402990
6	D009270	Naloxone	D007859	Learning Disorders	CTD/Clinical indications
7	D002927	Cimetidine	D006331	Heart Diseases	Clinical indications
8	D002927	Cimetidine	D007249	Inflammation	Clinical indications
9	D011239	Prednisolone	D008582	Meningitis	CTD/PMID: 33260200
10	D004294	Domperidone	D012640	Seizures	NA

**TABLE 5:** Top 10 drug-disease association prediction for dexamethasone and doxorubicin.

Drug Name	No.	MESH ID	Disease Name	Evidence
Dexamethasone MeSH ID: D003907	1	D004342	Drug Hypersensitivity	ClinicalTrials/CTD/PMID: 28704328
	2	D000743	Anemia, Hemolytic	CTD/PMID: 21848879
	3	D004417	Dyspnea	ClinicalTrials/PMID: 27330023
	4	D029424	Chronic Obstructive Pulmonary	Clinical indications
	5	D008581	Meningitis	NA
	6	D002637	Chest Pain	ClinicalTrials/CTD/PMID: 21799397
	7	D010190	Pancreatic Neoplasms	ClinicalTrials/CTD/PMID: 32619553
	8	D002318	Cardiovascular Diseases	NA
	9	D009205	Myocarditis	NA
	10	D012141	Respiratory Tract Infections	ClinicalTrials
Doxorubicin MeSH ID:D004317	1	D002289	Carcinoma, Non-Small-Cell Lung	ClinicalTrials/CTD/PMID: 33075540
	2	D014652	Vascular Diseases	ClinicalTrials/Clinical indications
	3	D009190	Myelodysplastic Syndromes	ClinicalTrials/CTD/PMID: 27299619
	4	D006463	Hemolytic-Uremic Syndrome	NA
	5	D002543	Cerebral Hemorrhage	NA
	6	D015473	Leukemia	ClinicalTrials/CTD/PMID: 32949646/Clinical indications
	7	D017202	Myocardial Ischemia	NA
	8	D011658	Pulmonary Fibrosis	ClinicalTrials/CTD/PMID: 22607134
	9	D050197	Atherosclerosis	NA
	10	D005910	Glioma	ClinicalTrials/CTD/PMID: 33475372

**TABLE 6:** Top 10 drug-disease association prediction for seizures and hypertension.

Disease Name	No.	MESH ID	Drug Name	Evidence
Seizures	1	D002034	Bumetanide	ClinicalTrials/CTD/PMID: 33201535
	2	D011239	Prednisolone	ClinicalTrials/CTD/PMID: 33359047
	3	D020123	Sirolimus	ClinicalTrials/CTD/PMID: 35931213
	4	C043211	Carvedilol	ClinicalTrials/CTD
	5	D013752	Tetracycline	ClinicalTrials/CTD/PMID:22579030
MeSH ID: D012640	6	D011802	Quinidine	ClinicalTrials/CTD/PMID: 30112700
	7	D014805	Vitamin B 12	ClinicalTrials/CTD/PMID: 29563977
	8	D013739	Testosterone	ClinicalTrials/CTD
	9	D017292	Doxazosin	NA
	10	D008691	Methadone	NA
Hypertension	1	C055162	Clopidogrel	ClinicalTrials/CTD/PMID: 35656824
	2	C060836	Pioglitazone	ClinicalTrials/CTD/PMID: 31712626
	3	C065180	Fluvastatin	ClinicalTrials/CTD/PMID: 17666915
	4	D000086	Acetazolamide	ClinicalTrials/PMID: 26154918
	5	D002738	Chloroquine	ClinicalTrials/CTD
MeSH ID: D006973	6	D004155	Diphenhydramine	ClinicalTrials
	7	D004958	Estradiol	NA
	8	D013629	Tamoxifen	ClinicalTrials/CTD
	9	D015283	Citalopram	NA
	10	D000068877	Imatinib Mesylate	ClinicalTrials/CTD

#### 3.5.1 Top 10 drug-disease associations

We listed the top 10 drug-disease associations predicted by LBMFF in Table 4, and eight out of them can be demonstrated by the four verification methods mentioned above. For example, we found evidence from public literature for rifampin combinations for treating pneumonia (PMID: 28870736), losartan for the prevention of paroxysmal atrial fibrillation in patients with sick sinus syndrome (PMID: 25787020) and simvastatin for improving the early survival rate of patients with pancreatic cancer (PMID: 32402990). As a prospective study, the combination therapy of prednisolone and azathioprine for steroid-responsive meningitis-arteritis treatment in dogs also appeared to be effective for primary treatment. Besides, several predictions have been confirmed effective by ClinicalTrials and CTD records, such as clopidogrel in patients with idiopathic pulmonary arterial hypertension and simvastatin in patients with advanced pancreatic cancer. We further verified three of the predictions have been applied as mature clinical treatments by passing clinical trials and safety tests. Naloxone is used to relieve respiratory depression and wake people up. Cimetidine is indicated for the treatment of arrhythmia and chronic hepatitis B hepatitis. This is consistent with the predicted treatment of heart disease and inflammation.

#### 3.5.2 Top 10 associated diseases for given drugs

**FIGURE 5:**
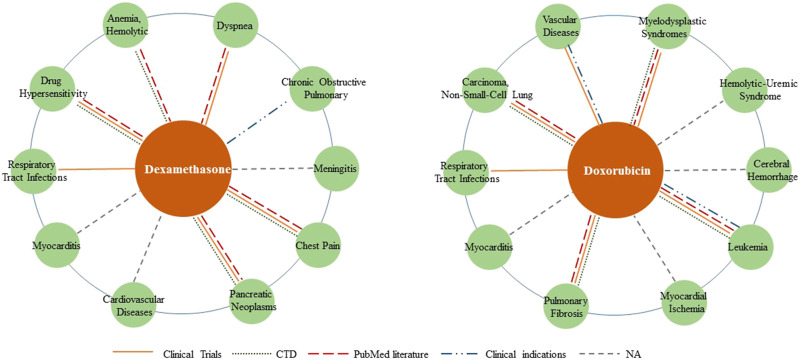
Top 10 associated diseases for given drugs. Different validation methods are represented with different colors and types of lines. The more lines between two nodes, the more evidences there are for this relationship.

We selected dexamethasone (MeSH ID: D003907) and doxorubicin (MeSH ID: D004317) as two drug cases to validate the ability to discover new indications. For each drug, the top 10 candidate diseases are ranked according to the prediction scores as shown in Table 5. We also visualized the predicted relationships (Figure 5) with different colors and types of lines to represent different validation methods. The more lines between two nodes, the more evidences there were for this relationship.

Dexamethasone is a corticosteroid that prevents the release of substances in the body that cause inflammation, such as allergic disorders and skin conditions. It is also used to treat ulcerative colitis, arthritis, lupus, psoriasis, and respiratory disorders. Seven of the top 10 predicted associations have been confirmed by databases, literature and clinical use of dexamethasone. According to literature, dexamethasone works against paclitaxel drug allergy (PMID: 28704328), chest syndrome in patients with sickle cell disease (PMID: 21799397) and dyspnea in cancer patients (PMID: 27330023). What’s more, WHO (2023) welcomes preliminary results about dexamethasone use in treating critically ill COVID-19 patients, as evidence of respiratory tract infection treatment.

Doxorubicin is an anthracycline type of chemotherapy that is used to treat several different types of cancer. Six of the top ten predicted associations have been confirmed in this section. It is approved for the treatment of non-small cell lung cancer, glioma, hematologic tumors and acute lymphoblastic leukemia, either alone or in combination with other drugs. Additionally, a combination of prednisone, azathioprine, and N-acetylcysteine (NAC) has also been used as a treatment for idiopathic pulmonary fibrosis (Behr, 2012).

The remaining associations predicted by the LBMFF model have not received much attention so far, providing an avenue for new indications to be discovered.

#### 3.5.3 Top 10 associated drugs for given diseases

**FIGURE 6:**
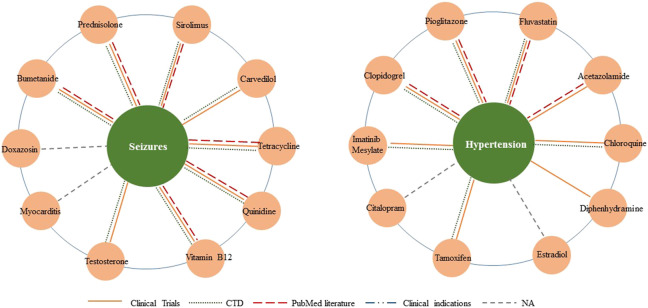
Top 10 associated drugs for given diseases. Different validation methods are represented with different colors and types of lines. The more lines between two nodes, the more evidences there are for this relationship.

Furthermore, we conducted two detailed case studies to further verify the capability of new therapies discovery, and the chosen diseases were seizures (MeSH ID: D012640) and hypertension (MeSH ID: D006973). The top 10 related drugs for both diseases were listed in Table 6. We also visualized the predicted relationships (Figure 6) with different colors and types of lines to represent different validation methods. The more lines between two nodes, the more evidences there were for this relationship.

In the section on seizures, we confirmed eight of the top 10 results through database and literature evidence. Specifically, quinidine significantly reduced the seizure burden (by about 90%). Tetracycline-class antibiotics were protective against partial seizures *in vivo*. The Drug combinations of bumetanide plus phenobarbital and vitamin B12 plus carbamazepine have been proven effective in treating seizures (PMID: 29563977). Moreover, animal models in mice presented with prednisolone or sirolimus had less severe seizures than the negative control group.

In the section on hypertension, we found evidence for eight drug candidates in the top ten through databases and literature. For example, pioglitazone modulated the vascular contractility in hypertension by interference with the ET-1 pathway (PMID: 31712626), and acetazolamide leads to more effective control of increased intracranial pressure (PMID: 26154918). Furthermore, ClinicalTrials and CTD proved the possibility of the other six new drug-disease associations.

## 4 Conclusion

In this study, we proposed a method called LBMFF for drug-disease association prediction. Due to the huge amount of information contained in both biomedical public databases and scientific literature, we computed drug-drug and disease-disease similarities by multi-feature fusion and utilized two GCN layers to capture structural embeddings from the association feature matrix. Concretely, the association feature matrix consisted a drug comprehensive similarity matrix, a disease comprehensive similarity matrix and a known drug-disease association. Moreover, an attention mechanism was denoted into the GCN model to extract information more effectively. The proposed method achieved excellent performance compared to seven state-of-the-art methods on the same test datasets, and we demonstrated its potential for identifying new drug-disease associations for practical use.

However, there are still some limitations in our work that require an in-depth investigation. First, more association features should be further considered in our work. We can collect more prior biological knowledge from literature, such as drug-protein, drug-gene, disease-gene and drug-pathway, to improve similarity accuracy. Second, the two-layer GCN is a basic model for learning on graph-structured data, while some other graph neural network models are worth investigating in the future.

Above all, LBMFF is able to learn scattered information from both public databases and scientific literature to identify the latent drug-disease associations. It gives researchers, pharmacologists, and pharmaceutical companies a tremendous opportunity to study and validate predictive associations that are more likely to exist. We expect LBMFF to be an efficient approach that can further improve drug repositioning and shorten its cost and time.

## Data Availability

The datasets presented in this study can be found in online repositories. The names of the repository/repositories and accession number(s) can be found in the article/supplementary material.
